# Unveiling the Role of Emotion Regulation in the Relationship between Intimate Partner Violence Increases and Post-Traumatic Stress Disorder: A Mediation Analysis

**DOI:** 10.3390/bs14090799

**Published:** 2024-09-10

**Authors:** Federica Taccini, Alessandro Alberto Rossi, Stefania Mannarini

**Affiliations:** 1 Department of Philosophy, Sociology, Education and Applied Psychology, Section of Applied Psychology, University of Padova, 35131 Padova, Italy; a.rossi@unipd.it (A.A.R.); stefania.mannarini@unipd.it (S.M.); 2 Center for Intervention and Research on Family Studies—CIRF, Department of Philosophy, Sociology, Education and Applied Psychology, Section of Applied Psychology, University of Padova, 35131 Padova, Italy

**Keywords:** intimate partner violence, trauma, emotion regulation, post-traumatic stress disorder

## Abstract

(1) Background: Experiencing intimate partner violence (IPV) can greatly impact victims’ physical and mental health, often leading to post-traumatic stress disorder (PTSD). Emotion regulation has been identified in the literature as a factor that contributes to the manifestation of PTSD. Consequently, this study aims to investigate the relationship among the increase in IPV victimization (i.e., physical, psychological, sexual, and economic violence), emotion dysregulation, and PTSD symptoms. It has been hypothesized that emotion dysregulation may mediate the increase in IPV occurrence and PTSD symptoms. (2) Methods: 284 women (M_age_ = 40.92) exposed to IPV were recruited in Italy. IPV experience was screened using the Revised Conflict Tactic Scale questionnaire. A mediational analysis was performed using Rstudio. (3) Results: The findings corroborated the mediating role of emotion dysregulation: the relationship between the increase in IPV and PTSD symptoms appears to be mediated by emotion dysregulation. (4) Conclusions: These findings bolster the existing literature regarding the association between emotion dysregulation and PTSD, underscoring the important role of emotion dysregulation in trauma symptoms. This highlights the significance of prioritizing the treatment of emotion dysregulation as a focal point for intervention and support for those who have experienced IPV.

## 1. Introduction

Intimate partner violence (IPV) refers to any form of violence that occurs within an intimate relationship, encompassing physical, psychological, sexual, and economic abuse [1]. Globally, about 27% (UI 23–31%) of women aged between 15 and 49, who have been married or in a partnership at some point in their lives, have experienced physical and/or sexual violence from a partner at least once [2]. The COVID-19 pandemic has caused several challenges to societies worldwide. Beyond its devastating health consequences, the pandemic has also unveiled a concerning rise in various social issues, including the increase in IPV occurrences [3,4,5,6,7]. In fact, the measures put into practice to mitigate the spread of the virus (e.g., lockdowns, social distancing) have inadvertently caused a situation where individuals subjected to IPV may have experienced close proximity to their perpetrators, increasing the risk of further acts of violence [8,9,10]. Extensive research has illuminated the prevalence and characteristics of IPV during this extraordinary period [9]. These studies indicate that violence experienced an upward trend amidst the challenges and adversities faced during this time (e.g., [9,11,12]). This study will examine various forms of IPV—including physical, psychological, sexual, and economic abuse—experienced by women and explore their relationship with post-traumatic stress disorder (PTSD), with a particular focus on the mediating role of emotion dysregulation.

In fact, the adverse impact of IPV on women’s mental well-being has been well documented, particularly concerning the emergence and persistence of PTSD [13,14,15,16,17,18]. In fact, the traumatizing nature of IPV experiences can elicit a cascade of cognitive, affective, and physiological reactions, which contribute to the development of post-traumatic stress symptoms (PTSSs). These manifestations encompass intrusive thoughts, avoidance behaviors, and hyperarousal [19,20,21,22]. Studies indicate that there may be many factors at play in the development of PTSD in individuals with an IPV experience. Among these there are the number of violent episodes and of abusive partners encountered, the timing of the IPV incidents, the presence of other life-threatening situations during the violent episodes, and the increase in violence occurrences [23,24]. Regarding the latter, the literature demonstrates that an increase in violence occurrence is closely associated with PTSD symptoms [25,26,27,28]. As the severity and frequency of various forms of IPV escalate, the likelihood of developing PTSD symptoms rises accordingly [25,26,27,28]. The experience of an increase in IPV episodes, however, is not an isolated phenomenon [29]: for example, a study by Krause et al. (2006) [30] found that around 36.7% of women reported IPV revictimization by the same partner within 12 months of seeking help for the violence. An increase in IPV also becomes a risk factor for further violent experiences, creating a vicious cycle that is even harder to break [31]. For instance, experiencing IPV appears to lead to the use of dysregulated emotion strategies and disengagement coping strategies (i.e., passive coping attempts, such as problem avoidance and social withdrawal), which, in turn, seem to contribute to further violent experiences [32,33].

Several factors have emerged as playing an important role in the relationship between the escalation of IPV and PTSS, such as insecure attachment [34], anger [35], and emotion regulation (e.g., [20,25,36]). Emotion dysregulation has been defined as the challenge in effectively managing and regulating one’s emotions [37]. A substantial body of literature has demonstrated the critical role of emotion regulation in individuals’ well-being and psychopathology [38,39,40]. Additionally, concerning women with an experience of IPV, various studies have emphasized the clinical benefits of focusing on this construct [41,42,43]. In fact, repeated exposure to acts of violence can disrupt an individual’s emotional regulation, leading to disordered emotional responses [20,44,45,46]. Such dysregulation may manifest in various ways, including intensified emotional responsiveness, challenges in recognizing emotions, limited access to strategies for regulating emotions, and a tendency to use maladaptive coping mechanisms [37,47,48]. An expanding body of literature has supported the mediating role of emotion dysregulation in the relationship between IPV and PTSD symptoms (e.g., [33,44,49,50]). Specifically, the occurrence of an increase in violence within an intimate relationship can result in elevated levels of emotion dysregulation, which subsequently contributes to the development and perpetuation of PTSD symptoms. For instance, in a recent study [44], the relationship among IPV, emotion dysregulation, and PTSS in women who experienced partner violence was investigated, demonstrating that emotion dysregulation of both negative and positive emotions mediates the association between various forms of IPV (physical, sexual, and psychological) and the severity of PTSS.

Nonetheless, the underlying processes connecting IPV, emotion dysregulation, and PTSD symptoms remain an area of interest and ongoing research. In fact, unraveling the mechanisms that link violence increases, emotion dysregulation, and PTSD symptoms may hold important implications for both research and clinical practice. In fact, identifying emotion dysregulation as a potential intermediary in the relationship between violence increases and PTSD symptoms can provide important insights for the development of targeted interventions that could alleviate the adverse effects of IPV. Interventions focused on enhancing emotion regulation have the potential to support women who have experienced IPV throughout their recovery journey by fostering healthier emotional reactions, diminishing the severity of symptoms, and enhancing overall well-being [49,51,52,53].

The objective of this research is to investigate the relationship among the increase in IPV encountered amidst the COVID-19 pandemic, emotion dysregulation, and PTSD symptoms in a sample of women with an experience of IPV. In this regard, the following hypothesis was formulated: emotion dysregulation may statistically significantly mediate the relationship between the increase in IPV and the manifestation of PTSD symptoms.

## 2. Materials and Methods

### 2.1. Procedure

This research involved the recruitment of several Anti-Violence Centres (AVCs) and shelters in Italy. From these AVCs and shelters, a total of 291 women who had experienced IPV during the COVID-19 pandemic were approached for participation in the research project. However, 7 participants decided not to continue, resulting in a final sample of 284 participants. The recruitment process involved contacting participants individually, and each participant completed the research survey individually.

The present study included individuals who met the following criteria: (a) age above 18; (b) native Italian speakers; (c) experienced IPV since the COVID-19 pandemic; (d) having accessed the AVC in the last two months. To assess the IPV experience of each recruited woman, the Revised Conflict Tactic Scale-2 (R-CTS2; Straus et al., 1996 [54]) was used, which evaluates violence experienced within the past year (see the measures section for more information on the self-report). Only women identified as having an IPV experience through the questionnaire were included in the study. Exclusion criteria consisted of the following: (e) difficulties in completing the battery of self-reports due to illiteracy, or (f) cognitive and/or visual difficulties.

The present research involved participants who willingly volunteered and expressed their informed consent. Approval for the research project was granted by the Ethics Committee of the University of Padua (protocol n° 4300). Participants, when agreeing to participate, were free to withdraw from the study at any time, and both the principal investigator of the present study and psychologists from each AVC recruited remained available during data collection to address any possible needs of the participants.

### 2.2. Participants

The group of participants included 284 women who had encountered IPV throughout the COVID-19 pandemic. The women in the group ranged in age from 20 to 71 years, with an average age of 40.92 (SD = 10.88). Regarding education, the breakdown of participants was as follows: 42 women (14.8%) had finished middle school, 158 women (55.6%) possessed a high school diploma, 35 women (12.3%) held a bachelor’s degree, 32 (11.3%) had a master’s degree, and 17 women (6.0%) had achieved a doctoral degree. In terms of their employment status, the majority of women, specifically 183 (64.3%), held regular jobs. A smaller portion of the participants, amounting to 41 (14.4%), were engaged in precarious employment. Out of those, 40 (14.1%) were unemployed, 15 (5.3%) were students, and 5 (1.8%) women were retired.

### 2.3. Measures

Demographic data were gathered, encompassing age, educational attainment, and employment status.

#### 2.3.1. Revised Conflict Tactic Scale (CTS2)

The Revised Conflict Tactics Scale (CTS2) [54,55], a questionnaire consisting of 39 items, was utilized as a screening tool to assess experiences of IPV. Its purpose was to gauge and evaluate the occurrence and intensity of physical, sexual, and psychological violence within a romantic relationship over the previous 12 months (e.g., “My partner shouted or yelled at me”). This questionnaire comprises five subscales, which all presented good internal reliability in the present study: physical violence (α = 0.90), psychological violence (α = 0.79), sexual violence (α = 0.53), injury (α = 0.57), and negotiation (α = 0.85), which assesses actions aimed at resolving disagreements through dialogue. Participants rate the frequency of each behavior using an 8-point scale, ranging from “never” to “more than 20 times”. Higher scores indicate a greater frequency of violent episodes. The Italian version of the questionnaire was employed [55]. 

#### 2.3.2. The Perception of Increase of Occurrences of Intimate Partner Violence (PI-IPV) 

The Perception of Increase of occurrences of Intimate Partner Violence (PI-IPV) [56] questionnaire was specifically created to investigate women’s perception of the increase of IPV occurrences within the past 12 months (e.g., “Have you experienced an increase in physical violence occurrences against you by your partner within the past 12 months?”). This measurement consists of four items. Participants provide ratings from “not at all” to “very much”. Higher scores on the scale indicate a higher increase in IPV. The internal consistency of the PI-IPV instrument was calculated for the purpose of the present study, and it was found to be satisfactory: α = 0.70.

#### 2.3.3. Difficulties in Emotion Regulation Scale-Short Form (DERS-SF)

The Short Form of the Difficulties in Emotion Regulation Scale (DERS-SF) [37,57] was used. The questionnaire includes 18 items (e.g., “I am clear about my feelings”) and utilizes a 5-point answering, ranging from “almost never” to “almost always”. The DERS-SF consists of a total scale (α = 0.81) and 6 subscales: (1) challenges in pursuing goal-oriented behaviors (α = 0.87), (2) non-acceptance of emotions (α = 0.77), (3) difficulties in impulsive control (α = 0.87), (4) limited awareness of emotions (α = 0.76), (5) restricted access to emotion regulation strategies (α = 0.81), and (6) lack of emotional clarity (α = 0.77). All the scales have shown great internal consistency in the present study. Higher scores indicate a greater frequency of challenges in emotion regulation. The Italian version of the DERS-SF was employed in this study [58]. In line with other studies (e.g., [56]), in this research, only the total scale score was used. 

#### 2.3.4. Impact of Event Scale Revised (IES-R)

The IES-R [59,60] is a questionnaire consisting of 22 items that rely on self-reporting. Its purpose is to evaluate the posttraumatic symptoms in individuals who have undergone a traumatic experience (e.g., “Any reminders brought back feelings about it”). It presents three subscales, which refer to the major symptom clusters of PTSD: hyperarousal, intrusion, and avoidance. Participants are asked to rate each item based on a 5-point answer, ranging from “not at all” to “extremely.” The questionnaire demonstrated strong internal consistency in the present study across various dimensions: the total score (α = 0.88), hyperarousal (α = 0.79), avoidance (α = 0.82), and intrusion (α = 0.93) [61]. In the present study, the total score was used in line with previous research (e.g., [62,63]).

### 2.4. Data Analysis

Rstudio [64,65] was used to conduct all the statistical analyses. The following packages were used: lavaan [66,67], lm.beta [68], psych [69], tidyverse [70], magrittr [71], dplyr [72], and GPA rotation [73].

Descriptive statistics are provided in Table 1 [74,75], and preliminary analyses were conducted before testing the mediation model. Correlation analyses were performed to detect possible cases of multicollinearity by utilizing the Spearman correlation coefficient due to non-normal distribution observed in certain variables. In this context, correlation coefficients higher than |0.80| were regarded as suggestive of multicollinearity [74,76].

In addition, in line with other studies (e.g., [56]), a multivariate multiple regression analysis was employed to account for potential factors, allowing for the examination of different forms of violence (such as psychological, physical, and sexual violence, as well as injuries and negotiation) as predictors, while psychological variables (specifically PTSD Symptoms and Emotion Dysregulation) were treated as the dependent variables. The standardized beta coefficients (β*) were used to assess the extent of influence of each predictor.

A mediational analysis was performed utilizing observed variables to investigate the direct influence of a predictor (escalation of IPV) on an outcome variable (PTSD symptoms). Additionally, the analysis explored the indirect impact of the predictor on PTSD symptoms through Emotion Dysregulation. The statistical analyses employed the maximum likelihood estimator (ML), and the goodness of fit of the model to the data was assessed using the Satorra-Bentler scaled χ^2^ [77]. The text contains unstandardized regression coefficients (ß), while Table 2 displays the standardized regression coefficients (ß*).

### 2.5. Sample Size Determination

The present study’s sample size was predetermined based on the planned statistical analysis for the current research (refer to the Data Analysis section). The study utilized the “n:q criterion” [75], where “n” represents the number of participants and “q” represents the number of model paths. To meet the minimum sample size requirement, it is advised to have a ratio of at least 30 participants per path (i.e., a 30:1 ratio) [75]. Consequently, a total of 90 participants were required for the current investigation.

## 3. Results

### 3.1. Preliminary Analysis

Descriptive statistics are present in Table 1. Correlation analyses (refer to Table 1) revealed statistically significant relationships among the variables considered in the mediation analysis. Nevertheless, none of the variables surpassed the recommended threshold of |0.80|, which means that statistical analyses could be conducted.

**Table 1:** Descriptive statistics and correlation among variables.

		Descriptive Statistics				Correlations							
		M	SD	SK	K	1	2	3	4	5	6	7	8
1	Psychological Aggression	39.890	38.380	0.930	0.019	-							
2	Sexual Coercion	5.316	11.337	2.744	7.366	0.422 **	-						
3	Physical Assault	7.954	20.408	6.062	53.020	0.606 **	0.459 **	-					
4	Injury	3.288	8.408	4.531	27.111	0.504 **	0.355 **	0.668 **	-				
5	Negotiation	29.246	34.731	1.489	1.671	0.051 §	0.088 §	0.133 *	0.091 §	-			
6	PI–IPV	6.581	3.926	0.621	0.449	0.418 **	0.295 **	0.410 **	0.342 **	−0.211 **	-		
7	DERS Total	45.419	14.228	0.522	−0.318	0.198 **	0.154 **	0.134 *	0.112 §	−0.021 §	0.207 **	-	
8	IES Total	34.133	9.936	−0.081	−0.040	0.258 **	0.218 **	0.225 **	0.226 **	−0.102	0.287 **	0.416 **	-

Note: ** *p* < 0.01; * *p* < 0.050; § *p* > 0.050 ns; SD = standard deviation; SK = skewness; K = Kurtosis; Psychological Aggression: Subscale of the Psychological Aggression of the Revised Conflict Tactic Scale; Sexual Coercion: Subscale of the Sexual Coercion of the Revised Conflict Tactic Scale; Physical Assault: Subscale of the Physical Assault of the Revised Conflict Tactic Scale; Injury: Subscale of the Injury of the Revised Conflict Tactic Scale; Negotiation (CTS2): Subscale of the Negotiation of the Revised Conflict Tactic Scale; PI-IPV: Total Scale of The Perception of Increase of occurrences of Intimate Partner Violence; DERS Total: Total Scale of the Difficulties of Emotion Regulation Scale; IES Total: Total Scale of the Impact of Event Scale.

A multivariate multiple regression analysis was performed to address potential confounding variables. The findings indicated that the predictors (covariates) had no statistically significant impact on the dependent variables (refer to Table 2 for standardized values). More specifically, even after accounting for other predictors, the variable (1) “Psychological Aggression (CTS2)” did not statistically significantly predict “Emotion Dysregulation (DERS Total)” (β = 0.044, SE = 0.027, *p* = 0.101). However, a statistically significant relationship was found with “PTSD Symptoms (IES Total)” (β = 0.040, SE = 0.018, *p* = 0.027).

In addition, (2) “Sexual Coercion (CTS2)” did not statistically significantly predict “PTSD Symptoms (IES Total)” (β = 0.167, SE = 0.085, *p* = 0.052). However, a statistically significant relationship was found with “Emotion Dysregulation (DERS Total)” (β = 0.180, SE = 0.057, *p* = 0.001).

Similarly, (3) “Physical Assault (CTS2)” showed no statistically significant relationship with “Emotion Dysregulation (DERS Total)” (β = 0.005, SE = 0.060, *p* = 0.927) and “PTSD Symptoms (IES Total)” (β = −0.003, SE = 0.040, *p* = 0.931).

In addition, (d) “Injury (CTS2)” showed no statistically significant relationship with “Emotion Dysregulation (DERS Total)” (β = 0.016, SE = 0.141, *p* = 0.907) and “PTSD Symptoms (IES Total)” (β = 0.033, SE = 0.095, *p* = 0.723).

Moreover, “Negotiation (CTS2)” did not statistically significantly predict “Emotion Dysregulation (DERS Total)” (β = 0.0003, SE = 0.024, *p* = 0.987). However, a statistically significant relationship was found with “PTSD Symptoms (IES Total)” (β = −0.025, SE = 0.016, *p* = 0.114).

These findings indicate that, after considering other factors, the predictors (external variables) did not have statistically significant effects on the dependent variables. The lack of significant effects suggests that the examined predictors do not play a substantial role in influencing the outcomes of interest.

**Table 2:** Multiple multivariate regression analysis among external variables and dependent variables involved in the mediation analysis.

External Variable	Dependent Variable	β*	β (SE)	95%CI [L, U]	*z*-Value	*p*-Value
Psychological Aggression	DERS Total	0.172	0.044 (0.027)	[−0.008; 0.098]	1.719	0.101
	IES Total	0.156	0.040 (0.018)	[0.004; 0.076]	2.181	0.027
Sexual Coercion	DERS Total	0.133	0.167 (0.085)	[−0.001; 0.335]	1.674	0.052
	IES Total	0.144	0.180 (0.057)	[0.067; 0.294]	3.589	0.001
Physical Assault	DERS Total	0.011	0.005 (0.060)	[−0.113; 0.124]	0.096	0.927
	IES Total	−0.007	−0.003 (0.040)	[−0.084; 0.077]	−0.074	0.931
Injury	DERS Total	0.009	0.016 (0.141)	[−0.261; 0.294]	0.109	0.907
	IES Total	0.019	0.033 (0.095)	[−0.154; 0.221]	0.362	0.723
Negotiation	DERS Total	0.001	0.0003 (0.024)	[−0.047; 0.048]	0.015	0.987
	IES Total	−0.090	−0.025 (0.016)	[−0.058; 0.006]	−1.208	0.114

Note: β* = standardized regression coefficient; β = unstandardized regression coefficient; SE = standard error; 95%CI = 95% confidence interval; Psychological Aggression: Subscale of the Psychological Aggression of the Revised Conflict Tactic Scale; Sexual Coercion: Subscale of the Sexual Coercion of the Revised Conflict Tactic Scale; Physical Assault: Subscale of the Physical Assault of the Revised Conflict Tactic Scale; Injury: Subscale of the Injury of the Revised Conflict Tactic Scale; Negotiation (CTS2): Subscale of the Negotiation of the Revised Conflict Tactic Scale; DERS Total: Total Scale of the Difficulties of Emotion Regulation Scale; IES Total: Total Scale of the Impact of Event Scale.

### 3.2. Mediation Model

The mediation model displayed positive indicators of goodness of fit.

**Figure 1:**
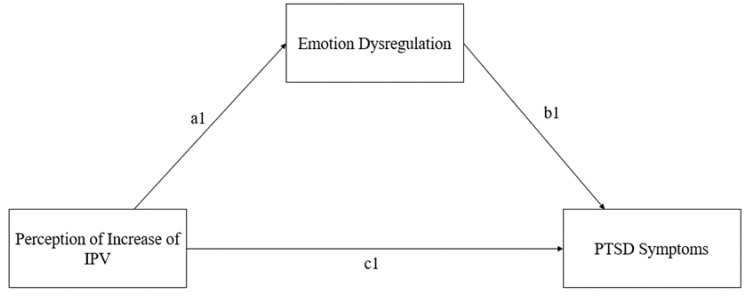
Model Conceptualization.

**Figure 2:**
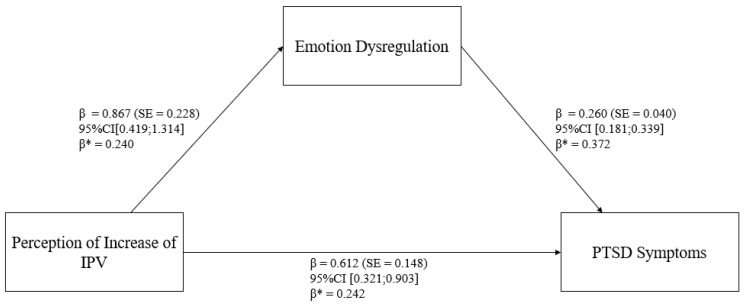
Statistical model.

In relation to the mediation model (refer to Table 3 and Figure 1 and Figure 2), the “Perception of Increase of IPV” (PI-IPV; X) was positively related to “Emotion Dysregulation” (DERS Total; M), path a1: β = 0.867 (SE = 0.228), z = 3.796, *p* < 0.001, 95% CI [0.419; 1.314]; β* = 0.240. The degree of explained variance was 5.7% (R^2^ = 0.057).

In addition, “Emotion Dysregulation” (DERS Total; M) statistically significantly predicted “PTSD Symptoms” (IES Total; Y), path b1: β = 0.260 (SE = 0.040), z = 6.455, *p* < 0.001, 95% CI [0.181; 0.339]; β* = 0.372. Furthermore, after controlling for “Emotion Dysregulation” (DERS Total; M), there was a statistically significant relationship between “Perception of Increase of IPV” and “PTSD Symptoms” (path c1: β = 0.612 (SE = 0.148), z = 0.413, *p* < 0.001, 95% CI [0.321; 0.903]; β* = 0.242). The degree of explained variance was 24.2% (R^2^ = 0.242).

The total indirect effect was as follows: β = 0.225 (SE = 0.069), z = 3.269, *p* = 0.001, 95% CI [0.090; 0.361]. Moreover, the total effect was as follows: β = 0.138 (SE = 0.043), z = 3.211, *p* = 0.001, 95% CI [0.054; 0.222].

## 4. Discussion

The aim of this study was to deepen our understanding of the relationship among the increase in violence occurrences during the COVID-19 pandemic, difficulties in regulating emotions, and PTSD symptoms in a sample of women who have experienced IPV. Understanding these relationships could contribute to provide valuable insights into the unique dynamics at play in the context of IPV during times of crisis.

First and foremost, it is crucial to highlight that the women who took part in this study disclosed an increase in the violent incidents during the COVID-19 pandemic. This reported upsurge in violence aligns with previous research, which has consistently indicated an increase in IPV cases amidst the COVID-19 outbreak [8,9,10]. The various stressors caused by the pandemic, including financial difficulties, and limited access to support networks, likely played an important role in increasing violence within intimate relationships. The findings from this study corroborate the existing literature, underscoring the alarming impact of the COVID-19 pandemic on the prevalence of IPV [3,9,11,78,79]. 

Furthermore, the results revealed a statistically significant relationship between the increase in IPV occurrences and emotion dysregulation. This may signify that as the severity or frequency of the IPV increase intensifies, a parallel rise in the participants’ experience of emotion dysregulation may be observed [37,49]. This outcome aligns with prior research, which proposes that the occurrence of increasing levels of IPV could hinder an individual’s capacity to manage their feelings, potentially resulting in emotional regulation difficulties [20,44,45,46]. For example, the results of the study by Villalta, et al. [80] show a statistically significant relationship between emotion dysregulation and PTSD symptoms in a sample of adolescents with an experience of sexual assault.

Additionally, the relationship between the increase in IPV and PTSD symptoms seems to be affected by the mediating role of emotion dysregulation. It is important to acknowledge that difficulties in emotion regulation have consistently been linked to various psychological disorders (e.g., anxiety, depression, and PTSS) [81,82,83,84,85,86]. For example, Weiss, Tull, Lavender, and Gratz [86] investigated the relationships among childhood abuse, emotion dysregulation, and probable PTSD in substance use disorder (SUD) patients receiving residential treatment. The results showed that difficulties in controlling impulsive behaviors when distressed were found to mediate the associations between childhood physical and emotional abuse and probable PTSD. 

Consistent with the aforementioned studies, the outcomes of the present research may support the significance of emotion dysregulation as a mediator in the association between the increase in IPV and PTSD symptoms. These findings highlight that individuals with elevated levels of an IPV increase may present an increased propensity to display challenges in controlling their feelings, which consequently may contribute to the formation or intensification of PTSD symptoms.

### 4.1. Clinical Implications

The implications of these results emphasize the potential importance of tackling emotion dysregulation as a main point for intervention among individuals experiencing an increase in IPV occurrences [87]. According to the literature [53,88], women who have experienced an increase in IPV occurrences and have been revictimized on several occasions might respond better to psychological interventions when emotion regulation difficulties are addressed directly. Consequently, experts should customize interventions to effectively tackle difficulties in regulating emotions. By promoting adaptive emotion regulation, individuals have the potential to witness enhancements in their mental well-being, resilience, and overall life satisfaction [20,89,90,91]. Several examples are present in literature based on interventions focused on emotion regulation for individuals exposed to trauma [87], such as W-ES.T.EEM [41], the *For Baby’s Sake* [42], and the STAIR [92]. Specifically, the W-ES.T.EEM intervention [41] for women exposed to IPV has three main modules. One of them is focused on emotion identification and regulation. Moreover, this module also aims to teach participants mindfulness and relaxation techniques. In addition, the *For Baby’s Sake* [42] intervention targets pregnant women exposed to IPV and their partner. The *For Baby’s Sake* [42] is delivered in modules. One of the modules is called “Healthy Expression of Feelings” and it consists of two parts: the first targets mothers and fathers’ emotion regulation strategies; the second aims at strengthening mothers and fathers’ emotional identification and expression in a healthy way. The STAIR [92] is a cognitive–behavioral intervention that focuses on emotion regulation and the improvement of interpersonal skills. It is based on the following topics: “(1) labeling and identifying feelings, (2) emotion management (particularly anger and anxiety), (3) distress tolerance, (4) acceptance of feelings and enhanced experiencing of positive emotions, (5) identification of trauma based interpersonal schemas and their enactment in day-to-day life, (6) identification of conflict between trauma-generated feelings and current interpersonal goals, (7) role plays related to issues of power and control, and (8) role plays related to developing flexibility in interpersonal situations involving power differentials” (p. 1069). In summary, these interventions offer techniques to enhance the awareness and tolerance of emotions through exercises, like assessing emotional states, improving emotion identification, and practicing breathing exercises. Additionally, victims may be guided to reconstruct their trauma narrative to eliminate self-blame. 

In conclusion, by focusing on emotion dysregulation, interventions can successfully strive towards reducing the detrimental effects of increasing occurrences of IPV on the development and intensity of PTSS and/or PTSD [29,71].

### 4.2. Limitations and Future Directions

Acknowledging certain limitations of the study and recognizing potential future research objectives is of utmost importance. First, it is critical to acknowledge that acts of violence can also be present in same-sex relationships [93,94,95]. Nevertheless, this study focused only on women’s encounters of IPV. Second, this investigation employed a cross-sectional approach. Consequently, establishing causality of the variables being examined [96] is not possible. Future studies could implement longitudinal designs to explore the nature of the relationships and acquire a more comprehensive understanding of the temporal linkages among the escalation of IPV, emotional dysregulation, and symptoms of PTSD. Third, the study relied on self-reported assessments, which might be susceptible to biases. Future studies could employ diverse approaches, such as interviews, to augment the validity of the findings. Four, participants’ experiences of other traumatic events that could have contributed to the PTSS were not pre-screened. Future studies should assess the experience of additional traumatic events as well. In addition, future studies could investigate the role of additional potential mediating variables that could interact in the relationship between escalating IPV and symptoms of PTSD, such as insecure attachment [34] and anger [35]. Additionally, future research should replicate these findings to validate this model in the absence of a global pandemic at the time of data collection. 

## 5. Conclusions

In conclusion, the results highlight that there was an increase in IPV during the COVID-19 pandemic. Moreover, the potential mediating role of emotion dysregulation in the relationship between IPV escalation and PTSD symptoms was shown. Consequently, emotion dysregulation should be a target for intervention in individuals exposed to IPV. In fact, interventions aimed at enhancing emotion regulation strategies can play a crucial role in mitigating the adverse impact of IPV and fostering psychological well-being among women with an IPV experience.

## Figures and Tables

**Table 3:** Mediation model coefficients.

Path		β*	β (SE)	95% CI [L-U]	*z*-Value	R^2^
Outcome: DERS Total (M) Perception of Increase of IPV (X) → DERS Total (M)	(a1)	0.240	0.867 (0.228)	[0.419; 1.314]	3.796 ***	0.057
Outcome: PTSD Symptoms (Y) DERS Total (M) → PTSD Symptoms (Y) Perception of Increase of IPV (X) → PTSD Symptoms (Y)	(b1) (c1)	0.372 0.242	0.260 (0.040) 0.612 (0.148)	[0.181; 0.339] [0.321; 0.903]	6.455 *** 0.413 ***	0.242
Effect of X on Y via M	(a1*b1)	0.089	0.225 (0.069)	[0.090; 0.361]	3.269 ***	
Total Effect of the Model		0.022	0.138 (0.043)	[0.054; 0.222]	3.211 ***	

Note: *** *p* < 0.001; DERS Total: Total Scale of the Difficulties of Emotion Regulation Scale; PI-IPV: Total Scale of The Perception of Increase of occurrences of Intimate Partner Violence; PTSD Symptoms: Total Scale of the Impact of Event Scale.

## Data Availability

Research data are not shared for ethical reasons.

## References

[1] Krug G.E., Dahlberg L.L., Mercy A.J., Zwi B.A., Lozano R. (2002). World Report on Violence and Health.

[2] WHO (2021). Global, Regional and National Prevalence Estimates for Intimate Partner Violence against Women and Global and Regional Prevalence Estimates for Non-Partner Sexual Violence against Women.

[3] Gosangi B., Park H., Thomas R., Gujrathi R., Bay C.P., Raja A.S., Seltzer S.E., Balcom M.C., McDonald M.L., Orgill D.P. (2021). Exacerbation of Physical Intimate Partner Violence during COVID-19 Pandemic. Radiology.

[4] Mannarini S., Balottin L., Munari C., Gatta M. (2017). Assessing conflict management in the couple: The definition of a latent dimension. Fam. J..

[5] Cunha O., Caridade S., de Castro Rodrigues A., Cruz A.R., Peixoto M.M. (2023). Perpetration of Intimate Partner Violence and COVID-19-Related Anxiety During the Second Lockdown in Portugal: The Mediating Role of Anxiety, Depression, and Stress. J. Fam. Violence.

[6] Ambrosetti J., Macheret L., Folliet A., Wullschleger A., Amerio A., Aguglia A., Serafini G., Prada P., Kaiser S., Bondolfi G. (2021). Psychiatric emergency admissions during and after COVID-19 lockdown: Short-term impact and long-term implications on mental health. BMC Psychiatry.

[7] Amerio A., Lugo A., Stival C., Fanucchi T., Gorini G., Pacifici R., Odone A., Serafini G., Gallus S. (2021). COVID-19 lockdown impact on mental health in a large representative sample of Italian adults. J. Affect. Disord..

[8] Kofman Y.B., Garfin D.R. (2020). Home is not always a haven: The domestic violence crisis amid the COVID-19 pandemic. Psychol. Trauma: Theory Res. Pract. Policy.

[9] Kourti A., Stavridou A., Panagouli E., Psaltopoulou T., Spiliopoulou C., Tsolia M., Sergentanis T.N., Tsitsika A. (2023). Domestic Violence During the COVID-19 Pandemic: A Systematic Review. Trauma Violence Abus..

[10] Mahase E. (2020). COVID-19: EU states report 60% rise in emergency calls about domestic violence. BMJ.

[11] Anurudran A., Yared L., Comrie C., Harrison K., Burke T. (2020). Domestic violence amid COVID-19. Int. J. Gynaecol. Obs..

[12] Barbara G., Facchin F., Micci L., Rendiniello M., Giulini P., Cattaneo C., Vercellini P., Kustermann A. (2020). COVID-19, Lockdown, and Intimate Partner Violence: Some Data from an Italian Service and Suggestions for Future Approaches. J. Womens Health.

[13] Trevillion K., Oram S., Feder G., Howard L.M. (2012). Experiences of Domestic Violence and Mental Disorders: A Systematic Review and Meta-Analysis. PLoS ONE.

[14] Bailey K., Trevillion K., Gilchrist G. (2019). What works for whom and why: A narrative systematic review of interventions for reducing post-traumatic stress disorder and problematic substance use among women with experiences of interpersonal violence. J. Subst. Abus. Treat..

[15] Beck J.G., McNiff J., Clapp J.D., Olsen S.A., Avery M.L., Hagewood J.H. (2011). Exploring negative emotion in women experiencing intimate partner violence: Shame, guilt, and PTSD. Behav. Ther..

[16] Beeble M.L., Bybee D., Sullivan C.M., Adams A.E. (2009). Main, mediating, and moderating effects of social support on the well-being of survivors of intimate partner violence across 2 years. J. Consult. Clin. Psychol..

[17] Nathanson A.M., Shorey R.C., Tirone V., Rhatigan D.L. (2012). The Prevalence of Mental Health Disorders in a Community Sample of Female Victims of Intimate Partner Violence. Partn. Abus..

[18] Taccini F., Mannarini S. (2024). How Are Survivors of Intimate Partner Violence and Sexual Violence Portrayed on Social Media?. J. Media Psychol..

[19] APA (2013). Diagnostic and Statistical Manual of Mental Disorders: DSM-5.

[20] Weiss N.H., Darosh A.G., Contractor A.A., Forkus S.R., Dixon-Gordon K.L., Sullivan T.P. (2018). Heterogeneity in emotion regulation difficulties among women victims of domestic violence: A latent profile analysis. J. Affect. Disord..

[21] Rossi A.A., Panzeri A., Taccini F., Parola A., Mannarini S. (2022). The Rising of the Shield hero. Development of the Post-Traumatic Symptom Questionnaire (PTSQ) and Assessment of the Protective Effect of self-esteem from trauma-related Anxiety and Depression. J. Child Adolesc. Trauma.

[22] Guglielmetti M., Serafini G., Amore M., Martelletti P. (2020). The relation between persistent post-traumatic headache and ptsd: Similarities and possible differences. Int. J. Environ. Res. Public Health.

[23] Bogat G.A., Levendosky A.A., Theran S., von Eye A., Davidson W.S. (2003). Predicting the psychosocial effects of interpersonal partner violence (IPV). How much does a woman’s history of IPV matter?. J. Interpers. Violence.

[24] Tran H.N., Beck J.G. (2019). Are Peritraumatic Perceptions of Fear/Life Threat and Posttraumatic Negative Self-Conscious Appraisals/Emotions Differentially Associated with PTSD Symptoms?. Cogn. Ther. Res..

[25] Ruork A.K., McLean C.L., Fruzzetti A.E. (2021). It Happened Matters More Than What Happened: Associations Between Intimate Partner Violence Abuse Type, Emotion Regulation, and Post-Traumatic Stress Symptoms. Violence Against Women.

[26] Jones L., Hughes M., Unterstaller U. (2001). Post-traumatic stress disorder (PTSD) in victims of domestic violence: A review of the research. Trauma Violence Abus..

[27] Becker K.D., Stuewig J., McCloskey L.A. (2010). Traumatic stress symptoms of women exposed to different forms of childhood victimization and intimate partner violence. J. Interpers. Violence.

[28] Chandra P.S., Satyanarayana V.A., Carey M.P. (2009). Women reporting intimate partner violence in India: Associations with PTSD and depressive symptoms. Arch. Women’s Ment. Health.

[29] Tomkins J., Jolliffe Simpson A.D., Polaschek D.L.L. (2023). High-risk Victims of Intimate Partner Violence: An Examination of Abuse Characteristics, Psychosocial Vulnerabilities and Reported Revictimization. J. Fam. Violence.

[30] Krause E.D., Kaltman S., Goodman L., Dutton M.A. (2006). Role of distinct PTSD symptoms in intimate partner reabuse: A prospective study. J. Trauma Stress.

[31] Kuijpers K.F., van der Knaap L.M., Winkel F.W. (2012). Risk of Revictimization of Intimate Partner Violence: The Role of Attachment, Anger and Violent Behavior of the Victim. J. Fam. Violence.

[32] Iverson K.M., Litwack S.D., Pineles S.L., Suvak M.K., Vaughn R.A., Resick P.A. (2013). Predictors of intimate partner violence revictimization: The relative impact of distinct PTSD symptoms, dissociation, and coping strategies. J. Trauma Stress.

[33] Lilly M.M., London M.J., Bridgett D.J. (2014). Using SEM to examine emotion regulation and revictimization in predicting PTSD symptoms among childhood abuse survivors. Psychol. Trauma Theory Res. Pract. Policy.

[34] Costa E.C.V., Botelheiro A.A.L.P. (2021). The impact of intimate partner violence on psychological well-being: Predictors of posttraumatic stress disorder and the mediating role of insecure attachment styles. Eur. J. Trauma Dissociation.

[35] Babcock J.C., Roseman A., Green C.E., Ross J.M. (2008). Intimate partner abuse and PTSD symptomatology: Examining mediators and moderators of the abuse-trauma link. J. Fam. Psychol. JFP J. Div. Fam. Psychol. Am. Psychol. Assoc. (Div. 43).

[36] Ehring T., Quack D. (2010). Emotion regulation difficulties in trauma survivors: The role of trauma type and PTSD symptom severity. Behav. Ther..

[37] Gratz K.L., Roemer L. (2004). Multidimensional Assessment of Emotion Regulation and Dysregulation: Development, Factor Structure, and Initial Validation of the Difficulties in Emotion Regulation Scale. J. Psychopathol. Behav. Assess..

[38] Kraiss J.T., ten Klooster P.M., Moskowitz J.T., Bohlmeijer E.T. (2020). The relationship between emotion regulation and well-being in patients with mental disorders: A meta-analysis. Compr. Psychiatry.

[39] Sloan E., Hall K., Moulding R., Bryce S., Mildred H., Staiger P.K. (2017). Emotion regulation as a transdiagnostic treatment construct across anxiety, depression, substance, eating and borderline personality disorders: A systematic review. Clin. Psychol. Rev..

[40] Verzeletti C., Zammuner V.L., Galli C., Agnoli S. (2016). Emotion regulation strategies and psychosocial well-being in adolescence. Cogent Psychol..

[41] Taccini F., Rossi A.A., Mannarini S. (2022). Women’s EmotionS, Trauma and EmpowErMent (W-ES.T.EEM) study protocol: A psychoeducational support intervention for victims of domestic violence—A randomised controlled trial. BMJ Open.

[42] Taccini F., Domoney J., Ocloo J., Heslin M., Byford S., Bick D., Howard L.M., MacMillan H., Mannarini S., Ramchandani P. (2024). ‘It’s so Beneficial to be Able to Stop the Cycle’: Perceptions of Intergenerational Transmission of Violence and Parenting Practices Among Pregnant Women and their Abusive Partners. J. Fam. Violence.

[43] Puente-Martínez A., Ubillos-Landa S., Rovira D.P. (2024). The mediating role of response-focused emotion regulation strategies in intimate partner violence across the stages of change. Curr. Psychol..

[44] Simpson L.E., Raudales A.M., Reyes M.E., Sullivan T.P., Weiss N.H. (2022). Intimate Partner Violence and Posttraumatic Stress Symptoms: Indirect Effects Through Negative and Positive Emotion Dysregulation. J. Interpers. Violence.

[45] Katz L.F., Gurtovenko K. (2015). Posttraumatic stress and emotion regulation in survivors of intimate partner violence. J. Fam. Psychol. JFP J. Div. Fam. Psychol. Am. Psychol. Assoc. (Div. 43).

[46] Goldsmith R.E., Chesney S.A., Heath N.M., Barlow M.R. (2013). Emotion Regulation Difficulties Mediate Associations Between Betrayal Trauma and Symptoms of Posttraumatic Stress, Depression, and Anxiety. J. Trauma Stress.

[47] Mannarini S., Balottin L., Palmieri A., Carotenuto F. (2018). Emotion regulation and parental bonding in families of adolescents with internalizing and externalizing symptoms. Front. Psychol..

[48] Miller M.W., Kaloupek D.G., Dillon A.L., Keane T.M. (2004). Externalizing and internalizing subtypes of combat-related PTSD: A replication and extension using the PSY-5 scales. J. Abnorm. Psychol..

[49] Weiss N.H., Nelson R.J., Contractor A.A., Sullivan T.P. (2019). Emotion dysregulation and posttraumatic stress disorder: A test of the incremental role of difficulties regulating positive emotions. Anxiety Stress Coping.

[50] Taylor S., Koch W.J., McNally R.J. (1992). How does anxiety sensitivity vary across the anxiety disorders?. J. Anxiety Disord..

[51] Iverson K., Shenk C., Fruzzetti A. (2009). Dialectical Behavior Therapy for Women Victims of Domestic Abuse: A Pilot Study. Prof. Psychol. Res. Pract..

[52] Katz L.F., Gurtovenko K., Maliken A., Stettler N., Kawamura J., Fladeboe K. (2020). An emotion coaching parenting intervention for families exposed to intimate partner violence. Dev. Psychol..

[53] Muñoz-Rivas M., Bellot A., Montorio I., Ronzón-Tirado R., Redondo N. (2021). Profiles of Emotion Regulation and Post-Traumatic Stress Severity among Female Victims of Intimate Partner Violence. Int. J. Environ. Res. Public Health.

[54] Straus M., Hamby S., Boney-McCoy S., Sugarman D. (1996). The Revised Conflict Tactics Scales (CTS2): Development and Preliminary Psychometric Data. J. Fam. Issues.

[55] Signorelli M., Arcidiacono E., Musumeci G., Di Nuovo S., Aguglia E. (2014). Detecting Domestic Violence: Italian Validation of Revised Conflict Tactics Scale (CTS-2). J. Fam. Violence.

[56] Taccini F., Rossi A.A., Mannarini S. (2024). Understanding the role of self-esteem and emotion dysregulation in victims of intimate partner violence. Fam. Process.

[57] Kaufman E.A., Xia M., Fosco G., Yaptangco M., Skidmore C.R., Crowell S.E. (2016). The Difficulties in Emotion Regulation Scale Short Form (DERS-SF): Validation and Replication in Adolescent and Adult Samples. J. Psychopathol. Behav. Assess..

[58] Rossi A.A., Panzeri A., Mannarini S. (2023). The Italian Version of the Difficulties in Emotion Regulation Scale—Short Form (IT-DERS-SF): A Two-step Validation Study. J. Psychopathol. Behav. Assess..

[59] Weiss D.S., Marmar C., Wilson J.P., Keane T. (1997). The Impact of Events Scale—Revised. Assessing Psychological Trauma and PTSD.

[60] Craparo G., Faraci P., Rotondo G., Gori A. (2013). The Impact of Event Scale—Revised: Psychometric properties of the Italian version in a sample of flood victims. Neuropsychiatr. Dis. Treat..

[61] Creamer M., Bell R., Failla S. (2003). Psychometric properties of the Impact of Event Scale—Revised. Behav. Res. Ther..

[62] Chew N.W.S., Lee G.K.H., Tan B.Y.Q., Jing M., Goh Y., Ngiam N.J.H., Yeo L.L.L., Ahmad A., Ahmed Khan F., Napolean Shanmugam G. (2020). A multinational, multicentre study on the psychological outcomes and associated physical symptoms amongst healthcare workers during COVID-19 outbreak. Brain Behav. Immun..

[63] Rodríguez-Rey R., Garrido-Hernansaiz H., Collado S. (2020). Psychological Impact and Associated Factors During the Initial Stage of the Coronavirus (COVID-19) Pandemic Among the General Population in Spain. Front. Psychol..

[64] R Core Team (2017). R: A Language and Environment for Statistical Computing.

[65] R Core Team (2014). The R Project for Statistical Computing.

[66] Rosseel Y. (2012). lavaan: An R package for structural equation modeling. J. Stat. Softw..

[67] Rosseel Y., Oberski D., Byrnes J., Vanbrabant L., Savalei V., Merkle E., Hallquist M., Rhemtulla M., Katsikatsou M., Barendse M. (2015). Package ‘lavaan’. https://cran.r-project.org/web/packages/lavaan/index.html.

[68] Backhaus K., Erichson B., Plinke W., Weiber R. (2010). Multivariate Analysemethoden. Eine Anwendungsorientierte Einführung. 11. Auflage.

[69] Revelle W. (2018). Psych: Procedures for Personality and Psychological Research. https://cran.r-project.org/web/packages/psych/index.html.

[70] Wickham H., Averick M., Bryan J., Chang W., McGowan L.D., François R., Grolemund G., Hayes A., Henry L., Hester J. (2019). Welcome to the tidyverse. J. Open Source Softw..

[71] Bache S., Wickham H. magrittr: A Forward-Pipe Operator for R. https://cran.r-project.org/web/packages/magrittr/magrittr.pdf.

[72] Wickham H., François R., Henry L., Müller K., Vaughan D. dplyr: A Grammar of Data Manipulation. https://dplyr.tidyverse.org.

[73] Bernaards C., Jennrich R. (2005). Gradient Projection Algorithms and Software for Arbitrary Rotation Criteria in Factor Analysis. Educ. Psychol. Meas..

[74] Hayes A.F. (2022). Introduction to Mediation, Moderation, and Conditional Process Analysis: A Regression-Based Approach.

[75] Kline R.B. (2016). Principles and Practice of Structural Equation Modeling.

[76] Tabachnick B.G., Fidell L.S. (2014). Using Multivariate Statistics.

[77] Satorra A., Bentler P.M. (2001). A scaled difference chi-square test statistic for moment structure analysis. Psychometrika.

[78] Peitzmeier S.M., Fedina L., Ashwell L., Herrenkohl T.I., Tolman R. (2022). Increases in Intimate Partner Violence During COVID-19: Prevalence and Correlates. J. Interpers. Violence.

[79] Moreira D.N., Pinto da Costa M. (2020). The impact of the Covid-19 pandemic in the precipitation of intimate partner violence. Int. J. Law Psychiatry.

[80] Villalta L., Khadr S., Chua K.C., Kramer T., Clarke V., Viner R.M., Stringaris A., Smith P. (2020). Complex post-traumatic stress symptoms in female adolescents: The role of emotion dysregulation in impairment and trauma exposure after an acute sexual assault. Eur. J. Psychotraumatol..

[81] Hechtman L.A., Raila H., Chiao J.Y., Gruber J. (2013). Positive Emotion Regulation and Psychopathology: A Transdiagnostic Cultural Neuroscience Approach. J. Exp. Psychopathol..

[82] Quoidbach J., Mikolajczak M., Gross J.J. (2015). Positive interventions: An emotion regulation perspective. Psychol. Bull..

[83] Abravanel B.T., Sinha R. (2015). Emotion dysregulation mediates the relationship between lifetime cumulative adversity and depressive symptomatology. J. Psychiatr. Res..

[84] Hofmann S.G., Sawyer A.T., Fang A., Asnaani A. (2012). Emotion dysregulation model of mood and anxiety disorders. Depress. Anxiety.

[85] Orgeta V. (2011). Emotion dysregulation and anxiety in late adulthood. J. Anxiety Disord..

[86] Weiss N.H., Tull M.T., Lavender J., Gratz K.L. (2013). Role of emotion dysregulation in the relationship between childhood abuse and probable PTSD in a sample of substance abusers. Child Abus. Negl..

[87] Siegel J.P. (2013). Breaking the Links in Intergenerational Violence: An Emotional Regulation Perspective. Fam. Process.

[88] Cloitre M., Scarvalone P., Difede J. (1997). Posttraumatic Stress Disorder, Self- and Interpersonal Dysfunction Among Sexually Retraumatized Women. J. Trauma. Stress.

[89] Bouthillier D., Julien D., Dubé M., Bélanger I., Hamelin M. (2002). Predictive Validity of Adult Attachment Measures in Relation to Emotion Regulation Behaviors in Marital Interactions. J. Adult Dev..

[90] Colombo D., Fernández-Álvarez J., García Palacios A., Cipresso P., Botella C., Riva G. (2019). New Technologies for the Understanding, Assessment, and Intervention of Emotion Regulation. Front. Psychol..

[91] Rossi A.A., Marconi M., Taccini F., Verusio C., Mannarini S. (2022). Screening for Distress in Oncological Patients: The Revised Version of the Psychological Distress Inventory (PDI-R). Front. Psychol..

[92] Cloitre M., Koenen K.C., Cohen L.R., Han H. (2002). Skills training in affective and interpersonal regulation followed by exposure: A phase-based treatment for PTSD related to childhood abuse. J. Consult. Clin. Psychol..

[93] Trombetta T., Rollè L. (2022). Intimate Partner Violence Perpetration Among Sexual Minority People and Associated Factors: A Systematic Review of Quantitative Studies. Sex. Res. Soc. Policy.

[94] Trombetta T., Balocco V., Santoniccolo F., Paradiso M.N., Rollè L. (2023). Internalized Homonegativity, Emotion Dysregulation, and Isolating Behaviors Perpetration among Gay and Lesbian Couples. Int. J. Environ. Res. Public Health.

[95] Mannarini S., Taccini F., Rossi A.A. (2023). The Role of Alexithymia and Impulsivity in Male Victims and Perpetrators of Intimate Partner Violence. Behav. Sci..

[96] Wang X., Cheng Z. (2020). Cross-sectional studies: Strengths, weaknesses, and recommendations. Chest.

